# Baseline assessment of cervical cancer screening and treatment capacity in 25 counties in Kenya, 2022

**DOI:** 10.3389/fonc.2024.1371529

**Published:** 2024-07-02

**Authors:** Valerian Mwenda, David Murage, Catherine Kilonzo, Joan-Paula Bor, Patricia Njiri, Lance Osiro, Mary Nyangasi, Marc Arbyn, Philippe Tummers, Marleen Temmerman

**Affiliations:** ^1^ National Cancer Control Program, Ministry of Health, Nairobi, Kenya; ^2^ Field Epidemiology and Laboratory Program, Ministry of Health, Nairobi, Kenya; ^3^ Noncommunicable Disease Program, Clinton Health Access Initiative, Nairobi, Kenya; ^4^ Unit of Cancer Epidemiology, Belgian Cancer Centre, Sciensano, Brussels, Belgium; ^5^ Department of Human Structure and Repair, Ghent University Hospital, Ghent, Belgium; ^6^ Cancer Research Institute Ghent (CRIG), Ghent, Belgium; ^7^ Department of Obstetrics and Gynaecology, Ghent University Hospital, Ghent, Belgium; ^8^ Department of Obstetrics and Gynaecology, Aga Khan University, Nairobi, Kenya

**Keywords:** cervical cancer, screening, Kenya, baseline assessment, service readiness

## Abstract

**Background:**

Cervical cancer is the leading cause of cancer deaths among women in Kenya. In the context of the Global strategy to accelerate the elimination of cervical cancer as a public health problem, Kenya is currently implementing screening and treatment scale-up. For effectively tracking the scale-up, a baseline assessment of cervical cancer screening and treatment service availability and readiness was conducted in 25 priority counties. We describe the findings of this assessment in the context of elimination efforts in Kenya.

**Methods:**

The survey was conducted from February 2021 to January 2022. All public hospitals in the target counties were included. We utilized healthcare workers trained in preparation for the scale-up as data collectors in each sub-county. Two electronic survey questionnaires (screening and treatment; and laboratory components) were used for data collection. All the health system building blocks were assessed. We used descriptive statistics to summarize the main service readiness indicators.

**Results:**

Of 3,150 hospitals surveyed, 47.6% (1,499) offered cervical cancer screening only, while 5.3% (166) offered both screening and treatment for precancer lesions. Visual inspection with acetic acid (VIA) was used in 96.0% (1,599/1,665) of the hospitals as primary screening modality and HPV testing was available in 31 (1.0%) hospitals. Among the 166 hospitals offering treatment for precancerous lesions, 79.5% (132/166) used cryotherapy, 18.7% (31/166) performed thermal ablation and 25.3% (42/166) performed large loop excision of the transformation zone (LLETZ). Pathology services were offered in only 7.1% (17/238) of the hospitals expected to have the service (level 4 and above). Only 10.8% (2,955/27,363) of healthcare workers were trained in cervical cancer screening and treatment; of these, 71.0% (2,097/2,955) were offering the services. Less than half of the hospitals had cervical cancer screening and treatment commodities at time of survey. The main health system strength was presence of multiple screening points at hospitals, but frequent commodity stock-outs was a key weakness.

**Conclusion:**

Training, commodities, and diagnostic services are major gaps in the cervical cancer program in Kenya. To meet the 2030 elimination targets, the national and county governments should ensure adequate financing, training, and service integration, especially at primary care level.

## Introduction

Cervical cancer is the second leading cause of cancer incidence and the leading cause of cancer deaths among women in Sub-Saharan Africa (SSA). In 2020, an estimated 117,316 cases of cervical cancer were diagnosed in Africa, and more than 76,000 women died from the disease in the continent, representing 22% of global deaths from cervical cancer (1). Majority of cervical cancer deaths occur among socio-economically disadvantaged women, especially those with poor access to quality health services (2, 3). While cervical cancer deaths continue falling in countries with organized screening programs and high human papillomavirus (HPV) vaccination coverage, the burden in SSA is increasing (4). In Kenya, cervical cancer is the leading cause of cancer deaths, with approximately 3,200 deaths reported in 2020 (1).

Cervical cancer has very effective modalities for screening, early diagnosis and treatment (5). To reduce the global burden of disease from cervical cancer, the World Health Organization (WHO) launched the Global strategy to accelerate the elimination of cervical cancer as a public health problem in 2020 (6). This strategy identifies key interventions and targets for countries globally by 2030: vaccination against HPV, screening with a high precision test and linkage to treatment. However, innovative strategies and collaborations are necessary to address low HPV vaccination coverage, low screening uptake and high loss to follow-up from screening programs, if low and middle-income countries are to move towards cervical cancer elimination (7). Health system strengthening and effective organization of cervical cancer screening programs have been identified as critical ingredients for success (8). Unfortunately, majority of SSA countries have not implemented and/or sustained high quality cervical cancer screening programs, due to health system deficiencies as well as socio-cultural influences (3, 9–11).

Cervical cancer screening coverage in Kenya was estimated at 16% in 2015 (12). One possible explanation for this low coverage is service availability; only a quarter of hospitals were offering cervical cancer screening services in 2018 (13). In order to move towards cervical cancer elimination, Kenya is implementing a national cervical cancer screening and treatment scale-up, targeting 25 priority counties since 2021. The scale-up involves healthcare workers training, supply of screening and treatment commodities and equipment as well as setting-up governance and coordination structures for the national cervical cancer program. Before the scale-up was launched, a baseline assessment of the cervical cancer screening and treatment service readiness was conducted in the 25 focus counties. The main objective of the baseline assessment was to provide an objective situational analysis of the national cervical cancer program, inform the planning of the scale-up and provide a basis for evaluating future successes of the targeted health system interventions. We present the findings from this assessment and its implications for cervical cancer elimination efforts in Kenya.

## Methods

### Study design and population

This was a cross-sectional survey, conducted in 25 of the 47 counties in Kenya, which were earmarked for the first phase of the national scale-up of cervical cancer screening and treatment. The counties were selected on the basis of HIV burden, regional representation, and sites where a previous pilot on cervical cancer screening scale-up had been carried out. The assessment was carried out over 12 months, from February 2021 to January 2022. The study population was hospitals, from level two (dispensaries) to level six (national referral hospitals) in the target counties. Screening using visual inspection with acetic acid (VIA), HPV sample collection, cryotherapy and thermal ablation are the modalities expected at level two and three hospitals; additional services like large loop excision of the transformation zone (LLETZ), HPV and cytology sample processing, biopsy and histology are expected from level four and above. All eligible hospitals in the selected counties were assessed. Two critical areas for cervical cancer screening programs were assessed in the hospitals: the screening service points and the laboratory. The specific areas assessed are shown in **Table 1**.

**Table 1:** Domains assessed at the hospitals during the study.

Domain	Items assessed
Hospital demographics	Name
	Sub-county
	County
	Level as per the Kenya Essential Package for Health (KEPH): level two (dispensary), three (health centre), four (sub-county hospital), five (county referral hospital) and six (national referral hospitals)
	Ownership: public, private, faith-based
	Catchment population
Service availability	Screening using visual inspection with acetic acid (VIA)
	Screening using human papillomavirus (HPV): sample collection
	Screening using HPV testing: sample processing
	Screening using cytology: sample collection
	Screening using cytology: sample processing
	Treatment: cryotherapy, thermal ablation, large loop excision of the transformation zone (LLETZ)
	Biopsy, endocervical curettage, colposcopy, histopathology Health products and supplies: acetic acid, cryotherapy gas, HPV, and pap smear kits
Service provision sites	Maternal and child health clinic
	Comprehensive care centres for HIV
	Outpatient department
	Gynaecologic clinic
	Theatre clinic
	Laboratory
Human resources for health	Number of healthcare workers per cadre, trained and/or deployed at cervical cancer screening and treatment service points
Minimum equipment for cervical cancer screening and treatment and commodities	White light source
	Examination room
	Examination couch
	HPV, cytology kits
	Acetic acid
	Applicator sticks
Infection prevention	Waste disposal bins
Awareness and advocacy	Methods used and frequency
Health information system	Electronic medical records systems (EMR), screening registers
Laboratory	Availability of a GeneXpert machine
	Sample referral mechanisms
	Backlogs
	Commodity stock outs

### Survey procedures

Two healthcare workers from each sub-county, who had already been identified and trained as peer trainers for cervical cancer screening and treatment, were utilized as data collectors for the survey. The trainers/data collectors had been selected from a pool of nurses/clinical officers/medical officers stationed at cervical cancer screening service provision points in their respective hospitals. A module on the survey tools and procedures was part of their training of trainers (TOT); it included administration of the questions, maneuvering through the electronic tools and data transmission procedures. This approach was deemed to be both efficient and provided an opportunity for the trainers to undertake hospitals mapping before they commenced their cascaded trainings. Each pair was then required to visit and administer the survey tools to hospitals managers, screening, and laboratory staff in all hospitals in their sub-county.

### Data collection and analysis

Data collection approaches included both interviewing key informants in various departments at the hospitals, as well as direct observation of hospitals/processes of interest. Data collection was conducted using two questionnaires: one for cervical cancer screening and treatment services and one for laboratory services. The questionnaires were created electronically using the SurveyCTO^©^ application and loaded into android tablets. Data was transmitted instantaneously to a central database, domiciled at the National Cancer Control Program (NCCP), for processing. Data cleaning and analysis were conducted using Epi-Info software (US CDC, Atlanta, GA). Descriptive statistics were calculated, in terms of the availability and readiness of various components of the cervical cancer screening and treatment program, across various strata including hospitals type and KEPH level.

## Findings

A total of 3,150 hospitals in 25 counties were assessed; majority 3,021 (95.9%) were public hospitals. Majority of the hospitals (3,122 [99.1%]) were primary health care hospitals (level 2-4). Cervical cancer screening was available in 1,665 hospitals (52.6%); however, only 166 (5.3%) were offering both screening and treatment for cervical cancer. The bulk of the health workforce available in the surveyed hospitals was made up of nurses (63.6% [18,639/29,326]). Awareness creation on cervical cancer screening services available was reported by 67.6% of the hospitals; (2,128/3,150); use of community health workers (86.2% [1,835/2,128]) and community outreaches (48.6% [1,035/2,128]) were the most popular methods for awareness creation (some facilities were using multiple approaches). Mass media was the least used approach (3.8%) even though it has the greatest capacity to reach many people. Clinical breast examination (CBE) was available at 78.3% (2,467/3,150) of the hospitals. Only 19.2% (606/3,150) had cervical cancer screening data capture and reporting tools at the time of the survey. Approximately 60% (1,905/3,150) of the hospitals had some form of EMR systems available at some service provision points; however, none had integrated cervical cancer screening data capture in the EMR. Other facility variables are shown in **Table 2**.

**Table 2:** Summary statistics of the hospitals surveyed.

Variable	Frequency	Proportion
Hospital tier (n=3,150)		
Level 2	2,264	71.9
Level 3	648	20.6
Level 4	210	6.7
Level 5	25	0.8
Level 6	3	0.1
Facility ownership (n=3,150)		
Public	3,021	95.9
Faith-based	69	2.2
Private/NGO	60	1.9
Cervical and breast cancer services offered (n=3,150)		
Awareness creation	2,128	67.6
Breast cancer screening	2,467	78.3
Cervical cancer screening only	1,499	47.6%
Cervical cancer screening and treatment	166	5.3%
Pathology (biopsy and histology)	17	0.5
Cadres of HCWs available (n=29,326)		
Nurses	18,639	63.6
Clinical officers	4,286	14.6
Laboratory technologists	3,050	10.4
Public Health Officers	1,935	6.6
Medical officers	1,215	4.1
Gynaecologists	134	0.5
Histo-technicians	28	0.1
Pathologists	20	0.1
Cytologists	19	0.1
Health information system (n=3,150)		
Data tools available	606	19.2
IEC materials available	774	24.6
Electronic health systems (n=1,905)		
EMR	240	12.6%
Internet	289	15.2%
Demand generation approaches (n=2,128)		
Cancer awareness months	540	25.4
Places of worship	432	20.3
Community Health Workers	1,835	86.2
Community Leaders	559	26.3
Mass media	80	3.8
Community Outreaches	1,035	48.6
Others	419	19.7

Some percentages may not be exactly 100% due to rounding-up to one decimal place.

### Service delivery per level of care

Cervical cancer screening service availability was highest at level 3 (70.5% [457/648]) and level 4 (67.1% [141/210]) (**Table 3**). However, availability of both screening and treatment was highest at level 5 hospitals (76% [19/25]). Majority of levels 2 and 3, which formed the bulk of the hospitals, did not have cervical pre-cancer treatment services.

**Table 3:** Screening and treatment service availability per facility level.

Facility Level	Number of hospitals (N)	Screening alone available n (%)	Both screening and pre-cancer treatment available n (%)
2	2,264	896 (39.6)	12 (0.5)
3	648	457 (70.5)	51 (7.8)
4	210	141 (67.1)	83 (39.5)
5	25	4 (16.0)	19 (76.0)
6	3	1 (33.3)	1 (33.3)
**Total**	3,150	1,499 (47.6)	166 (5.3)

**Figure 1:**
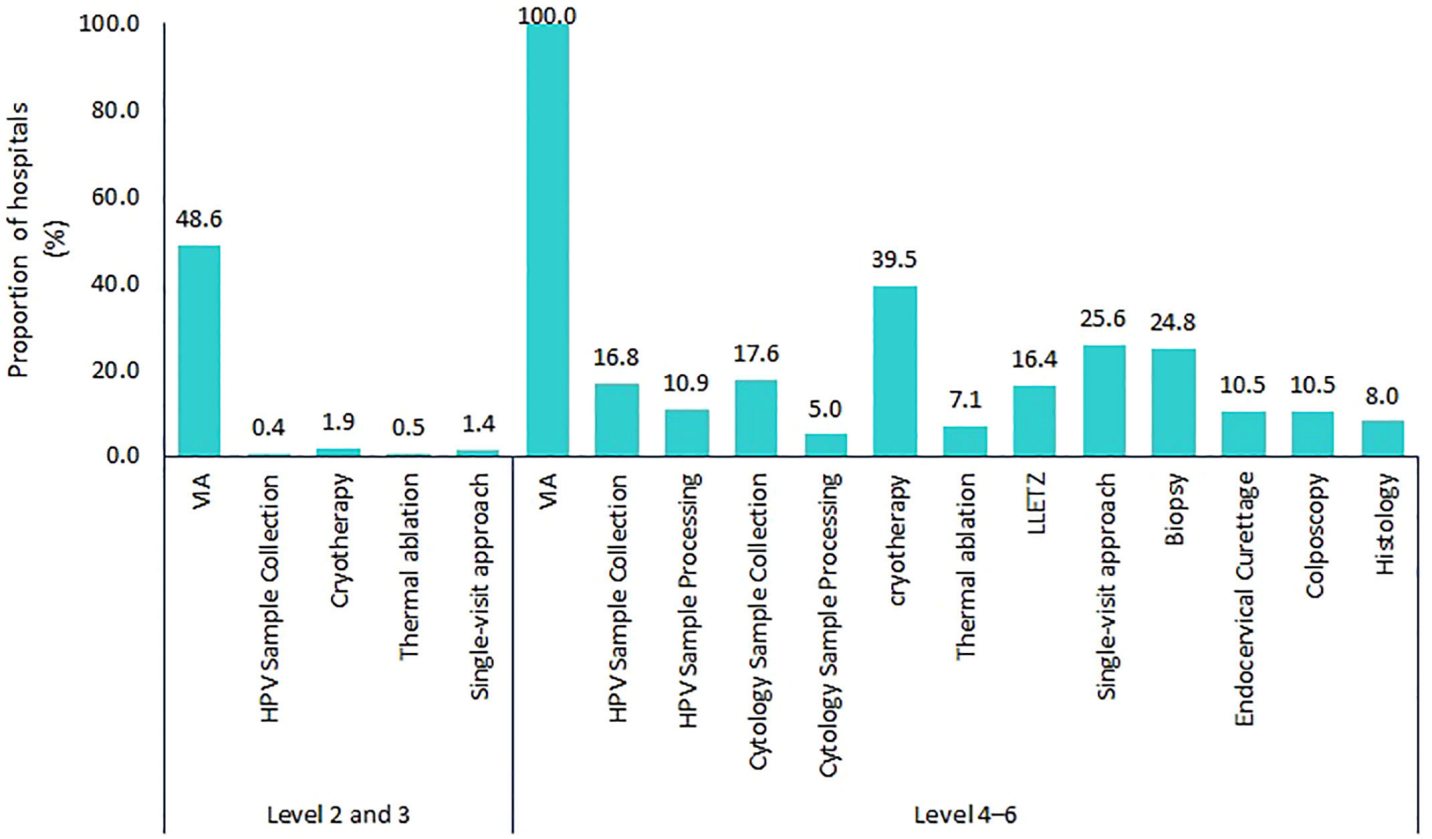
Proportion of assessed hospitals, offering various services along the cervical cancer screening and treatment continuum (level 2 and 3, n=2,912; level 4 and above, n=238). LLETZ: Large loop excision of the transformation zone; VIA: visual inspection with acetic acid. Single visit approach: both screening and treatment offered during the same visit.

The primary screening method used in most hospitals with screening services was VIA in 96.0% (1,599/1,665) of the hospitals. Among hospitals offering pre-cancer treatment, the modality commonly used was cryotherapy, available in 79.5% (132/166) of these hospitals; 63.9% (106/166) offered single visit approach. In diagnostics, cervical biopsy was available in 21.8% (52/238) of level four and above hospitals and histology in 7.1% (17/238). Among hospitals offering the service, the median cost of histopathology was $ 12.46 [IQR; 5.81–20.76]; the cost was borne by the patients in all the hospitals. Availability of other services across hospitals as per level of where the service is expected, is shown in **Figure 1**.

Most of the screening, diagnostic and treatment services were offered in the maternal and child health (MCH) clinic and comprehensive clinics (CCC) for people living with HIV; for instance, 66.5% (1,108/1,665) of the hospitals offering VIA were providing it at MCH only, 1.9% (32/1,665) at CCC alone and 24.7% (412/1,665) at both MCH and CCC.

### Screening and treatment health workforce

Only 10.8% (2,955/27,363) of all the HCWs were trained in cervical cancer screening and treatment, with nurses contributing 74.9% (2,212/2,955) of the trained workforce. Among those who are trained, 72.2% of nurses, 66.4% of clinical officers, 41.0% of medical officers, and 65.7% of gynecologists were deployed at cervical cancer screening and treatment service provision points at their hospitals (**Table 4**).

**Table 4:** Cervical cancer screening and treatment health workforce.

Cadre	Total number in the hospitals (N)	Number trained^*^ on cervical cancer screening n1 (n1/N %)	Number offering cervical cancer screening and treatment n2 (n2/n1%)
Nurses	18,639	2,212 (11.9)	1,598 (72.2)
Clinical officers	4,286	381 (8.9)	253 (66.4)
Medical officers	1,215	134 (11.0)	55 (41.0)
Gynecologists	134	108 (80.6)	71 (65.7)
Laboratory technologists	3,050	94 (3.1)	94 (100.0)
Pathologists	20	11 (55.0)	11 (100.0)
Cytologists	19	10 (52.6)	10 (100.0)
Histo-technologist	28	5 (17.9)	5 (100.0)

*Any form of focused training on cervical cancer screening and treatment, whether pre-service, formal, or on-job training in the previous three years.

### Screening commodities availability

**Figure 2:**
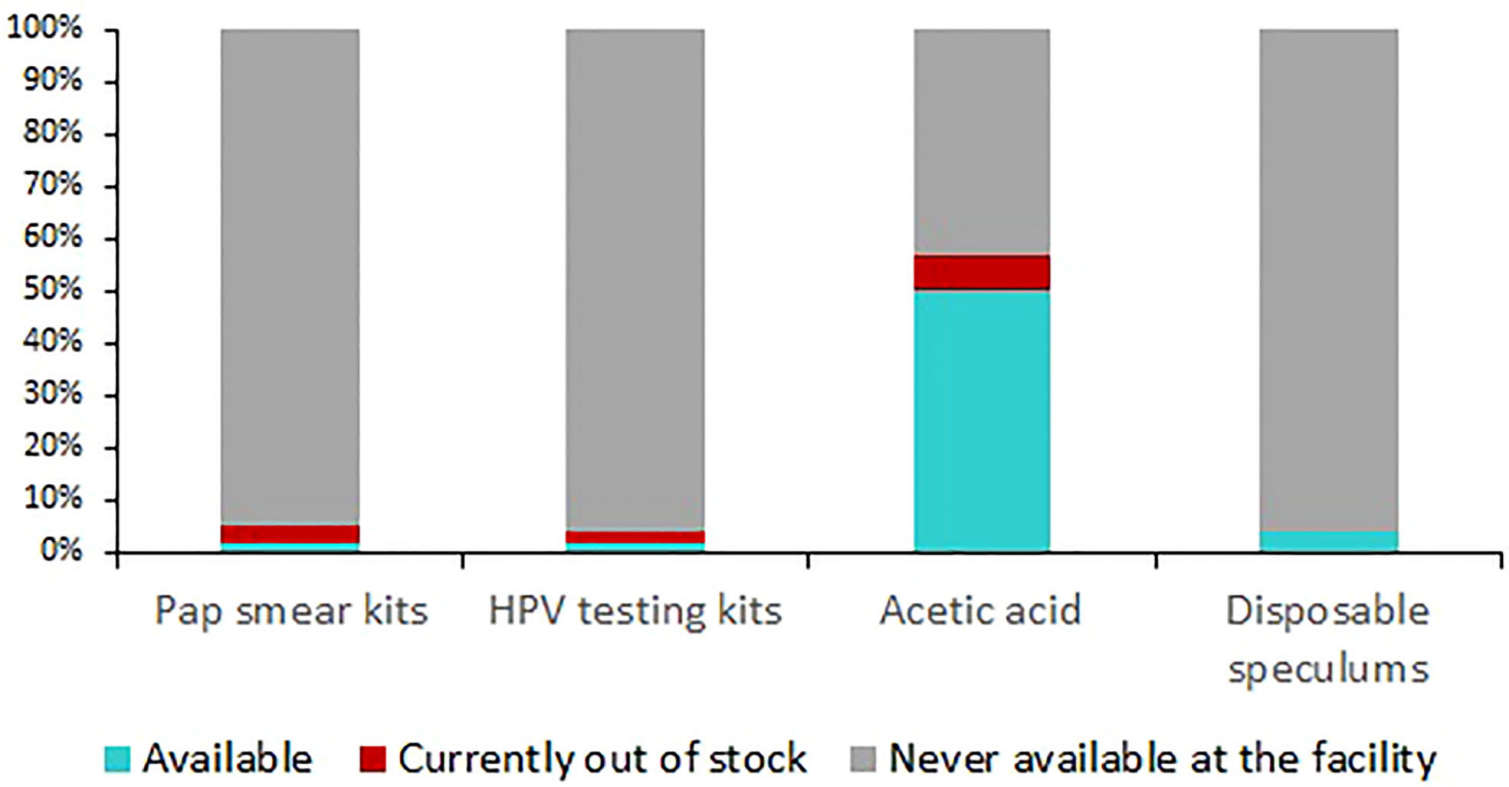
Availability of critical cervical cancer screening commodities in hospitals in 25 Kenyan counties, 2022. (n=3,150).

Among hospitals that had included cervical cancer screening in their service charter, half (830/1,665) had acetic acid available while 48.9% (815/1,665) had the recommended light source for pelvic examination at the time of the assessment. Other critical commodities like HPV tests and pap smear kits were available in less than five percent of the hospitals offering screening (**Figure 2**).

### Health system readiness

We noted some key strengths and weaknesses in the health system readiness in moving towards cervical cancer elimination (**Table 5**). Multiple service delivery points offer opportunities for a better reach and exploitation of efficiencies of service integration. Having multiple cadres offering cervical cancer screening and treatment offers a larger pool for service provision and skill-set strengthening for an effective cervical program. Cervical precancer lesions treatment availability is limited in the hospitals surveyed, which may reduce successful care linkage for women with positive screening results. Another major weakness is the erratic and inefficient supply chain for the screening and treatment commodities, especially cryotherapy gas that limited the number of hospitals able to offer both screening and treatment. Primary care hospitals offer free services, but are limited in service readiness for both screening and treatment.

**Table 5:** Strengths and weaknesses of the healthcare system to support cervical cancer screening and treatment in Kenya.

Health system building block	Strength	weakness
Leadership and governance	Some form of governance structure exists, with either reproductive health or non-communicable disease coordinator taking charge of cervical cancer screening and treatment planning at county level and sitting in the County Health Management Team.	Data-driven decision making has not been adequately embraced at facility and county level.
Service delivery	Services, where available, are spread out in multiple delivery points.	Treatment of cervical pre-cancerous lesions is available in very few hospitals. Primary health care hospitals, which constitute the majority of hospitals, have in sufficient service availability and readiness.
Health system financing	At health centre and dispensary level (level 2 and 3), cervical cancer screening and treatment is offered free of charge.	Cervical cancer screening is not covered under the National health Insurance Fund (NHIF); funding for screening is relegated to the background and priority given to curative programs. While screening is free at primary care hospitals (dispensaries and health centres), service provision is limited by trained workforce and health products stock-outs in these hospitals since they lack planning and budgeting autonomy.
Health workforce	Screening and treatment services are provided by multiple cadres, including nurses, clinical officers, medical officers, and gynaecologists.	High attrition rate of HCWs trained on cervical cancer screening and treatment makes it impossible to sustain highly trained and motivated teams.
Medical products, vaccines, and technologies	Most hospitals had the bare minimum screening commodities; speculums, gloves, and acetic acid.	Screening commodities supply is not prioritized, making it erratic and prone to frequent stock-outs. For instance, cryotherapy gas is commonly unavailable even where the equipment is available, therefore making many screening hospitals unbale to offer treatment.
Health information systems	A comprehensive cancer screening register has been developed and disseminated. Aggregated cervical cancer screening and treatment data is collected using primary and summary registers at facility level and uploaded into the Kenya Health Information System (DHIS2).	The paper-based system is inefficient in ensuring proper follow-ups and linkage to further evaluation/treatment. This is especially critical when clients with positive tests are referred for treatment in a different hospital.

## Discussion

### Summary of findings

We found that primary health care (PHC) hospitals form the bedrock of cervical cancer service provision in the 25 Counties surveyed. While more than half of all the hospitals offer cervical cancer screening, only 5.0% offer both screening and treatment. Only one in 10 of HCWs in the surveyed hospitals were trained in cervical cancer screening and treatment. Less than half of the hospitals had available stock of cervical cancer screening and treatment commodities at the time of the survey. Presence of multiple screening points at the hospitals was the main health system strength, but commodity stockouts was identified as the main weakness.

### Cervical cancer screening service readiness in Kenya

Majority of the surveyed hospitals were PHC level (2 and 3). This agrees with the structure of the overall health system in Kenya, where PHC hospitals form the bulk of the available public hospitals countrywide. Therefore, strengthening PHC system would be a major step in increasing access to cervical cancer screening and treatment, to make progress towards the 2030 elimination targets. Levels 2 and 3 also have service provision at no cost to patients, implying that they can be avenues for removing financial barriers to cervical cancer screening uptake. PHC, especially within the context of Universal Health Coverage (UHC), is important for increasing access to cervical screening (14).

Unfortunately, more than half of the PHC hospitals do not offer cervical cancer screening, and even those that do, fail to provide treatment. One reason may be inadequate trained and competent personnel; while some HCWs reported that they had received training in the past, some did not feel competent enough to offer treatment. Another reason could be erratic provision of screening and treatment health commodities and unavailability of treatment equipment. For instance, despite a country-wide distribution of cryotherapy equipment over a decade ago, we found that many were either broken, or had run out of cryotherapy gas and never replenished. PHC hospitals, while offering free services, have no financial planning autonomy, and rely on secondary level hospitals for procurement of supplies; in such circumstances health promotion interventions like cancer screening may be deprioritized when financial resources are very limited. Even where trained personnel were available at some point, they are lost by either transfer to other hospitals/departments or retirement from service and no regular replacements done. These findings are similar to a recent national service readiness survey in Kenya, which showed higher readiness in referral hospitals compared with PHC hospitals (15).

MCH and CCC/HIV clinics are the main cervical cancer screening service points in the surveyed hospitals. Traditionally, cervical cancer screening in Kenya was domiciled under the reproductive health services, hence services were offered either at MCH or family planning clinics. Organized cervical cancer screening also served as an integral component of HIV care, due to the epidemiological and biological linkage between HIV and cervical cancer. Integration is an efficient policy direction for increasing cervical cancer screening uptake; lessons from integration at MCH and CCC can enable incorporation of more service provision points at hospitals, including outpatient departments (OPD) and gynecological clinics. More hospitals were offering CBE than cervical cancer screening, proving another opportunity for integration. Ample evidence exists on the efficacy of integrating cervical cancer screening in reproductive, HIV and vaccination programs in SSA (16–23).

We found frequent unavailability of critical supplies for cervical cancer screening and treatment, especially acetic acid, cryotherapy gas and HPV kits. Procurement of such commodities may not be prioritized at the county level, compared with diagnostic commodities and medicines. In addition, screening commodities are not available at the Kenya Medical Supplies Authority (KEMSA), the main medical supplier for the County Departments of Health in Kenya, possibly due to policy or resource constraints. NHIF does not cover preventive or promotive health services like cancer screening, which severely limits the financing component of the national cervical cancer control program. However, this may change with the ongoing UHC reforms in the health sector. Lack of screening commodities was also identified as a key gap in an evaluation of the Zimbabwe cervical cancer program (24).

Multiple service provision points, by different cadres were identified as key strengths in the cervical cancer program in the surveyed hospitals; unavailability of treatment services, erratic commodity supply chain and few numbers of trained personnel were the major weaknesses. Availing multiple screening points at hospitals minimizes lost opportunities and increase screening uptake. Health service provision in Kenya is based on the Kenya Essential Package for Health (KEPH) levels; cervical cancer screening ideally is supposed to be offered across all the levels, but especially PHC hospitals (2–4). All the HCW cadres in these levels are eligible for training on cervical cancer screening and treatment, as guided by the respective schemes of service. Accessibility of screening and integrating with other services offered at the hospitals were noted as drivers of cervical cancer screening uptake in Malawi (25). In Uganda, building capacity among PHC health workers in cervical cancer screening and treatment has been adopted as a strategy to address unmet needs in the population (26). In addition to commodities supply chain, the Zimbabwean study also identified staffing challenges, lack of equipment, limited funding and ineffective leadership and governance structure (24). A similar approach, including training PHC personnel, adapting screening approaches to practical local contexts and enhancing local infrastructure to perform various screening tests, has been suggested for two West-African countries (27).

### Strengths and limitations

A particular strength of this study was that we conducted a census of all the hospitals in the 25 Counties, spread out in the 10 regions of Kenya; therefore, the findings are likely representative of the true state of cervical cancer control service readiness. The assessment also comprehensively examined the main health system building blocks, therefore provides critical insights for areas in need of strengthening for Kenya to move towards elimination. A weakness of the study was that the survey did not undertake an exploratory angle, to find out the possible underlying reasons to some of the identified gaps. Such an undertaking would have provided more information for planning and focusing the interventions in a more effective and efficient manner and is planned for subsequent program evaluations.

## Conclusion and recommendations

We identified major gaps in the service readiness for an effective cervical cancer program in the 25 Counties, but also some opportunities, which if explored can provide a path towards elimination. We recommend a more efficient supply for cervical cancer screening and treatment commodities at PHC, primarily through public financing. Since level 2 and 3 hospitals constitute the majority of the hospitals, they should be enabled to offer cervical cancer screening and treatment by ensuring adequately trained staff and essential health commodities. Availability of screening services in nearby hospitals has been identified as one of the determinants of screening uptake (28). Additional service provision points at hospitals need to integrate cervical cancer screening to their routine service provision, to reduce missed opportunities for screening when women visit for other services. A study in Ethiopia identified restricting screening to a single service point as a barrier to screening uptake (29). A cervical cancer human resource development plan is necessary to guide recruitment, training, mentorship, retention, and replacement of personnel at the county level; sustained capacity-building of HCWs is necessary for success of programs (30). Cervical cancer screening and treatment should be included in the ongoing health financing reforms, especially at PHC; recent evidence shows adequate financing will be necessary for cervical cancer elimination (31). Regular similar assessments should be conducted to inform the efficacy of ongoing investments in the strengthening of the national cervical cancer control program.

## Data availability statement

The original contributions presented in the study are included in the article/**Supplementary Material**. Further inquiries can be directed to the corresponding author.

## Ethics statement

Since the unit of assessment was the hospitals, we collected data on service availability and readiness; no personal information was obtained. The study was approved by the Ministry of Health (MoH) and the respective County Departments of Health. Specifically, the assessment was modelled on a broader health system service availability and readiness assessment, usually conducted for all health services in Kenya every five years. However, in this case, we focused on readiness for the country to implement selected interventions within the cervical cancer elimination strategy. Being a routine component of health system evaluation and strengthening, this assessment did not require an Institutional Review Board (IRB) clearance process as per relevant stipulations by the Ministry of Health. All methods were carried out in accordance with the Monitoring and Evaluation guidelines of MoH. Even though the data collected was not personal in nature, safety was ensured during transmission and archiving through password-protected files.

## Author contributions

VM: Conceptualization, Data curation, Formal analysis, Investigation, Methodology, Project administration, Resources, Software, Supervision, Validation, Visualization, Writing – original draft, Writing – review & editing. DM: Formal analysis, Methodology, Validation, Writing – original draft, Writing – review & editing. CK: Formal analysis, Writing – original draft, Writing – review & editing. J-PB: Conceptualization, Investigation, Validation, Writing – original draft, Writing – review & editing. PN: Conceptualization, Funding acquisition, Project administration, Resources, Supervision, Validation, Writing – original draft, Writing – review & editing. LO: Conceptualization, Investigation, Validation, Writing – original draft, Writing – review & editing. MN: Conceptualization, Funding acquisition, Investigation, Project administration, Supervision, Writing – original draft, Writing – review & editing. MA: Writing – original draft, Writing – review & editing. PT: Writing – original draft, Writing – review & editing. MT: Writing – original draft, Writing – review & editing.

## Supplementary material

Data Sheet 1 Screening and treatment services assessment dataset.

Data Sheet 2 Laboratory services assessment dataset.

Data Sheet 3 Screening and treatment services assessment codebook.

Data Sheet 4 Laboratory services assessment dataset codebook.

## References

[1] Sung H Ferlay J Siegel RL Laversanne M Soerjomataram I Jemal A. Global cancer statistics 2020: GLOBOCAN estimates of incidence and mortality worldwide for 36 cancers in 185 countries. CA Cancer J Clin. (2021) 71:209–49. doi: 10.3322/caac.21660 33538338

[2] WHO. Address inequality: prevent cervical cancer. Available online at: https://www.who.int/mediacentre/commentaries/cervical-cancer-prevention/en/.

[3] Temmerman M Bustreo F. Cervical cancer services are the next frontier for universal healthcare coverage in LMICs. BMJ opinion blogs. (2017). Available: https://blogs.bmj.com/bmj/2017/09/20/cervical-cancer-services-are-the-next-frontier-for-universal-healthcare-coverage-in-lmics/.

[4] Jedy-Agba E Joko WY Liu B Buziba NG Borok M Korir A. Trends in cervical cancer incidence in sub-Saharan Africa. Br J Cancer. (2020) 123:148–54. doi: 10.1038/s41416-020-0831-9 PMC7341858 32336751

[5] Ngoma M Autier P. Cancer prevention: cervical cancer. Ecancermedicalscience. (2019) 13:952. doi: 10.3332/ecancer.2019.952 31552125 PMC6722108

[6] WHO. A Global Strategy for elimination of cervical cancer. Available online at: https://www.who.int/activities/a-global-strategy-for-elimination-of-cervical-cancer.

[7] Shin MB Liu G Mugo N Garcia PJ Rao DW Bayer CJ. A framework for cervical cancer elimination in low-and-middle-income countries: A scoping review and roadmap for interventions and research priorities. Front Public Health. (2021) 9:670032. doi: 10.3389/fpubh.2021.670032 34277540 PMC8281011

[8] Bhatla N Nessa A Oswal K Vashist S Sebastian P Basu P. Program organization rather than choice of test determines success of cervical cancer screening: Case studies from Bangladesh and India. Int J Gynaecol Obstet. (2021) 152:40–7. doi: 10.1002/ijgo.13486 33205399

[9] Pierz AJ Randall TC Castle PE Adedimeji A Ingabire C Kubwimana G. A scoping review: Facilitators and barriers of cervical cancer screening and early diagnosis of breast cancer in Sub-Saharan African health settings. Gynecol Oncol Rep. (2020) 33:100605. doi: 10.1016/j.gore.2020.100605 32637528 PMC7327246

[10] Yimer NB Mohammed MA Solomon K Tadese M Grutzmacher S Meikena HK. Cervical cancer screening uptake in Sub-Saharan Africa: a systematic review and meta-analysis. Public Health. (2021) 195:105–11. doi: 10.1016/j.puhe.2021.04.014 34082174

[11] Lim JN Ojo AA. Barriers to utilisation of cervical cancer screening in Sub Sahara Africa: a systematic review. Eur J Cancer Care. (2017) 26:1–9. doi: 10.1111/ecc.2017.26.issue-1 26853214

[12] Ng'ang'a A Nyangasi M Nkonge NG Gathitu E Kibachio J Gichangi P. Predictors of cervical cancer screening among Kenyan women: results of a nested case-control study in a nationally representative survey. BMC Public Health. (2018) 18:1221. doi: 10.1186/s12889-018-6054-9 30400916 PMC6219012

[13] Ministry of Health. Kenya hospitals assessment report, 2019. Available online at: http://www.health.go.ke/wp-content/uploads/2020/01/KHFA-2018-19-Popular-version-report-Final-.pdf.

[14] Woo YL Gravitt P Khor SK Ng CW Saville M. Accelerating action on cervical screening in lower- and middle-income countries (LMICs) post COVID-19 era. Prev Med. (2021) 144:106294. doi: 10.1016/j.ypmed.2020.106294 33678225 PMC7931730

[15] Ammoun R Wami WM Otieno P Schultsz C Kyobutungi C Asiki G. Readiness of hospitals to deliver non-communicable diseases services in Kenya: a national cross-sectional survey. BMC Health Serv Res. (2022) 22:985. doi: 10.1186/s12913-022-08364-w 35918710 PMC9344761

[16] Dreyer G Botha MH Snyman LC Visser C Burden R Laubscher N. Combining cervical cancer screening for mothers with schoolgirl vaccination during human papillomavirus (HPV) vaccine implementation in South Africa: results from the VACCS1 and VACCS2 trials. Int J Gynecol Cancer. (2022) 32:592–8. doi: 10.1136/ijgc-2021-003079 35078829

[17] Pfaff C Singano V Akello H Amberbir A Berman J Kwekwesa A. Early experiences in integrating cervical cancer screening and treatment into HIV services in Zomba Central Hospital, Malawi. Malawi Med J. (2018) 30:211–4. doi: 10.4314/mmj.v30i3.14 PMC6307048 30627358

[18] Davies NECG Chersich M Mullick S Naidoo N Makhoba N Rees H. Integrating Cervical Cancer Screening into Safer Conception Services to Improve Women's Health Outcomes: A Pilot Study at a Primary Care Clinic in South Africa. Sex Transm Dis. (2019) 46:91–7. doi: 10.1097/OLQ.0000000000000914 PMC6336485 30308532

[19] Tchounga B Boni SP Koffi JJ Horo AG Tanon A Messou E. Cervical cancer screening uptake and correlates among HIV-infected women: a cross-sectional survey in Côte d'Ivoire, West Africa. BMJ Open. (2019) 9:e029882. doi: 10.1136/bmjopen-2019-029882 PMC6720463 31473620

[20] Diala PC Randa M Odhiambo J Ganda G Cohen CR Mungo C. Barriers and facilitators to integrating clinical breast examinations with cervical cancer screening programs in outpatient clinics in Western Kenya. JCO Glob Oncol. (2021) 7:1722–9. doi: 10.1200/GO.21.00272 PMC8710349 34936373

[21] Wirtz C Mohamed Y Engel D Sidibe A Holloway M Bloem P. Integrating HPV vaccination programs with enhanced cervical cancer screening and treatment, a systematic review. Vaccine. (2022) 40 Suppl 1:A116–23. doi: 10.1016/j.vaccine.2021.11.013 34863615

[22] Castle PE Einstein MH Sahasrabuddhe VV. Cervical cancer prevention and control in women living with human immunodeficiency virus. CA Cancer J Clin. (2021) 71:505–26. doi: 10.3322/caac.21696 PMC10054840 34499351

[23] Leno DWA Diallo FD Delamou A Komano FD Magassouba M Niamy D. Integration of family planning counselling to mass screening campaign for cervical cancer: experience from Guinea. Obstet Gynecol Int. (2018) 2018:3712948. doi: 10.1155/2018/3712948 29713347 PMC5866874

[24] Tapera O Nyakabau AM Simango N Guzha BT Jombo-Nyakuwa S Takawira E. Gaps and opportunities for cervical cancer prevention, diagnosis, treatment and care: evidence from midterm review of the Zimbabwe cervical Cancer prevention and control strategy (2016-2020). BMC Public Health. (2021) 21:1478. doi: 10.1186/s12889-021-11532-y 34320957 PMC8318330

[25] Pittalis C Panteli E Schouten E Magongwa I Gajewski J. Breast and cervical cancer screening services in Malawi: a systematic review. BMC Cancer. (2020) 20:1101. doi: 10.1186/s12885-020-07610-w 33183270 PMC7663900

[26] Jatho A Mugisha NM Kafeero J Holoya G Okuku F Niyonzima N. Capacity building for cancer prevention and early detection in the Ugandan primary healthcare hospitals: Working toward reducing the unmet needs of cancer control services. Cancer Med. (2021) 10:745–56. doi: 10.1002/cam4.3659 PMC7877353 33319508

[27] Haque A Kouriba B Aïssatou N Pant A. Eliminating cervical cancer in Mali and Senegal, two sub-Saharan countries: insights and optimizing solutions. Vaccines (Basel). (2020) 8:181. doi: 10.3390/vaccines8020181 32295116 PMC7349839

[28] Atnafu DD Khatri R Assefa Y. Drivers of cervical cancer prevention and management in sub-Saharan Africa: a qualitative synthesis of mixed studies. Health Res Policy Syst. (2024) 22:21. doi: 10.1186/s12961-023-01094-3 38331830 PMC10851545

[29] Jemal Z Chea N Hasen H Tesfaye T Abera N. Cervical cancer screening utilization and associated factors among female health workers in public hospitals of Hossana town, southern Ethiopia: A mixed method approach. PloS One. (2023) 18:e0286262. doi: 10.1371/journal.pone.0286262 37252937 PMC10228814

[30] Moucheraud C Kawale P Kafwafwa S Bastani R Hoffman RM. Health care workers' experiences with implementation of "screen and treat" for cervical cancer prevention in Malawi: A qualitative study. Implement Sci Commun. (2020) 1:112. doi: 10.1186/s43058-020-00097-3 33317633 PMC7734769

[31] Financing for cervical cancer elimination. UICC. (2023) Available at: https://www.uicc.org/what-we-do/thematic-areas-work/cervical-cancer-elimination/financing-cervical-cancer-elimination.

