# Supplementary material for: Baseline assessment of cervical cancer screening and treatment capacity in 25 counties in Kenya, 2022
Source: Front Oncol. 2024 Jul 2;14:1371529. doi: 10.3389/fonc.2024.1371529 (PMC11249718; doi:10.3389/fonc.2024.1371529)
Supplement: Data Sheet 3 — Screening and treatment services assessment codebook. [file DataSheet_3.pdf]

Cervical Cancer Facility Baseline Assessment

Field	Question	Answer
General Information		
name (required)	Name of facility incharge	
tel (required)	Telephone<br>Format: 7xxxxxxxx	
email (required)	Email	
date	Date of supervision	
superteam	Supervision Team Members(Format:Name-Designation)<br>Hint:Grace Wanjiku-Medical Officer	
super1	1	
super2	2	
super3	3	
respo.	Respondents(Format:Name-Designation)<br>Hint:Tom Oduor-Nurse	
respo1	1	
respo2	2	
respo3	3	
County (required)	Please select your County	County County
Sub (required)	Please select the name of your Sub-County	Sub Sub
Facility (required)	Please select the name of your facility	Facility Facility
ftier (required)	Facility Level Tier	1 Level 1<br>2 Level 2<br>3 Level 3<br>4 Level 4<br>5 Level 5<br>6 Level 6
ownership (required)	Facility Ownership	1 GOK<br>2 Private<br>3 NGO<br>4 FBO
wrapop (required)	Facility Catchment Population(WRA)	
note1	The following areas will be assessed:Service Availability,Inputs and Processes	
Service Availability		
serv1 (required)	Does the facility service charter include provision of cervical cancer screening and treatment?<br>Check service charter and confirm the specific services indicated...	1 Yes<br>0 No<br>3 To be updated
serv2 (required)	What is the indicated waiting time?<br>Hint:Indicate waiting time in minutes e.g 10 minutes	
serv3	Does this facility provide any cervical cancer screening services?<br>Select yes if they provide at least one service	1 Yes<br>0 No
serv3a	Does this facility provide any cervical cancer treatment services?<br>Select yes if they provide at least one service	1 Yes<br>0 No
note2	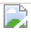<br>Please swipe left to select the cervical cancer screening and treatment services currently being provided.	
Services Provided		
labels	Service	1 Yes<br>0 No
cyts (required)	Cytology(Sample Collection)	1 Yes<br>0 No
cytp (required)	Cytology(Processing)	1 Yes<br>0 No
hpvs (required)	HPV Test(Sample Collection)	1 Yes<br>0 No
hpvp (required)	HPV Test(Processing)	1 Yes<br>0 No
via (required)	VIA	1 Yes<br>0 No
vili (required)	VILI	1 Yes<br>0 No

Field	Question	Answer
cry (required)	Cryotherapy	1 Yes<br>0 No
sva (required)	Single Visit Approach(Screen & Treat)	1 Yes<br>0 No
leep (required)	LEEP	1 Yes<br>0 No
col (required)	Colposcopy	1 Yes<br>0 No
bio (required)	Biopsy	1 Yes<br>0 No
endo (required)	Endocervical Curettage	1 Yes<br>0 No
hist (required)	Histology/Pathology	1 Yes<br>0 No
ther (required)	Thermoablation	1 Yes<br>0 No
Place of Service Provision		
labels2	Place of Service Provision	1 MCH<br>2 CCC<br>3 Gynae Clinic<br>4 Theatre Clinic<br>5 Lab
cyts2 (required)	Cytology(Sample Collection)	1 MCH<br>2 CCC<br>3 Gynae Clinic<br>4 Theatre Clinic<br>5 Lab
cytp2 (required)	Cytology(Processing)	1 MCH<br>2 CCC<br>3 Gynae Clinic<br>4 Theatre Clinic<br>5 Lab
hpvs2 (required)	HPV Test(Sample Collection)	1 MCH<br>2 CCC<br>3 Gynae Clinic<br>4 Theatre Clinic<br>5 Lab
hpvp2 (required)	HPV Test(Processing)	1 MCH<br>2 CCC<br>3 Gynae Clinic<br>4 Theatre Clinic<br>5 Lab
via2 (required)	VIA	1 MCH<br>2 CCC<br>3 Gynae Clinic<br>4 Theatre Clinic<br>5 Lab
vili2 (required)	VILI	1 MCH<br>2 CCC<br>3 Gynae Clinic<br>4 Theatre Clinic<br>5 Lab
cry2 (required)	Cryotherapy	1 MCH<br>2 CCC<br>3 Gynae Clinic<br>4 Theatre Clinic<br>5 Lab

Field	Question	Answer
sva2 (required)	Single Visit Approach(Screen & Treat)	1 MCH<br>2 CCC<br>3 Gynae Clinic<br>4 Theatre Clinic<br>5 Lab
leep2 (required)	LEEP	1 MCH<br>2 CCC<br>3 Gynae Clinic<br>4 Theatre Clinic<br>5 Lab
col2 (required)	Colposcopy	1 MCH<br>2 CCC<br>3 Gynae Clinic<br>4 Theatre Clinic<br>5 Lab
bio2 (required)	Biopsy	1 MCH<br>2 CCC<br>3 Gynae Clinic<br>4 Theatre Clinic<br>5 Lab
endo2 (required)	Endocervical Curettage	1 MCH<br>2 CCC<br>3 Gynae Clinic<br>4 Theatre Clinic<br>5 Lab
hist2 (required)	Histology/Pathology	1 MCH<br>2 CCC<br>3 Gynae Clinic<br>4 Theatre Clinic<br>5 Lab
ther2 (required)	Thermoablation	1 MCH<br>2 CCC<br>3 Gynae Clinic<br>4 Theatre Clinic<br>5 Lab
Facility Readiness		
doc (required)	Number of medical officers in health facility	
doccax (required)	Number of medical officers trained in cervical cancer screening and treatment	
docdeplo	Number of medical officers trained and deployed in cervical cancer screening and treatment area	
doctrain (required)	Type of training	1 Pre-Service Training<br>2 In-Service Training(OJT)<br>3 Formal Training
nurse (required)	Number of nurses in health facility	
nursecax (required)	Number of nurses trained in cervical cancer screening and treatment	
nursedeplo (required)	Number of trained nurses deployed in the cervical cancer screening and treatment area	
nursetrain (required)	Type of training	1 Pre-Service Training<br>2 In-Service Training(OJT)<br>3 Formal Training
co (required)	Number of clinical officers in health facility	
cocax (required)	Number of clinical officers trained in cervical cancer screening and treatment	
codeplo (required)	Number of clinical officers trained and deployed in cervical cancer screening and treatment area	
cotrain (required)	Type of training	1 Pre-Service Training<br>2 In-Service Training(OJT)<br>3 Formal Training
labtech (required)	Number of lab.techs in health facility	
labtechcax (required)	Number of lab.techs trained in cervical cancer screening and treatment	
gyn (required)	Number of gynaecologist in health facility	
gyncax (required)	Number of gynaecologists trained in cervical cancer screening and treatment	
gyndeplo (required)	Number of gynaecologists trained and deployed in cervical cancer screening and treatment area	
path (required)	Number of pathologists in health facility	

Field	Question	Answer
pathcax (required)	Number of pathologists trained in cervical cancer screening and treatment	
cyt (required)	Number of cytologist in health facility	
cytcax (required)	Number of cytologists trained in cervical cancer screening and treatment	
histo (required)	Number of histotechnicians in health facility	
histcax (required)	Number of histotechnicians trained in cervical cancer screening and treatment	
facility1		
comms (required)	Do you employ community strategy (involving PHOs,CHVs) in the implementation of cervical cancer screening and treatment activities in your facility?	1 Yes<br>0 No
facility2		
noPHOs (required)	Number of PHOs attached to the facility	
noCHVs (required)	Number of CHVs attached to the facility	
pho (required)	Are PHOs and CHVs attached to your facility sensitized on cervical cancer screening and treatment?	1 Yes<br>0 No
facility3		
path1 (required)	Does the facility offer cervical cancer pathology services?	1 Yes<br>0 No
facility4		
path1a (required)	Where are the cervical cancer pathology samples processed?	1 Processed at the facility LAB<br>2 Referred to an external LAB
facility5		
paps1 (required)	Where are the cervical cancer pathological (PAP smear) samples referred to ?	1 Private LAB<br>2 Private referral LAB<br>3 Public referral county facility LAB<br>4 Public referral national LAB<br>5 Other
facility6		
refferal (required)	Does the facility have a formal arrangement with the referral facility ?	1 Yes<br>0 No
paps2 (required)	Do you refer ;	1 Sample Only<br>2 Client together with the sample
cacxcost (required)	How much does it cost to process a cervical cancer sample ?<br>KSHS.	
cacxcost1 (required)	Who bears the cost of pathology?	1 Client<br>2 Facility<br>3 Cost Sharing<br>4 Other
PAPTAT (required)	What is the turnaround time for PAP smear results?	1 < 2 weeks<br>2 2 to 4 weeks<br>3 > 4 weeks
Availability of Guidelines		
guide1 (required)	Does the facility have the current national cervical cancer screening and treatment guidelines(2018)?	1 Yes<br>0 No
Infrastructure		
infra1 (required)	Is there a space/room available for cervical cancer screening services ?	1 Yes<br>0 No
Infrastructure		
infra2 (required)	Where can such a room be established?	
Screening Equipment/Items		
labels4	Item Availability	1 Yes<br>0 No
exambed (required)	Examination Bed	1 Yes<br>0 No
lights (required)	Light Source(White Light)	1 Yes<br>0 No
Current Stock Status		
labels5	Stock Status	1 Available<br>2 Out of Stock(< 1 month)<br>3 Out of Stock(> 1 month)

Field	Question	Answer
		4 Not Available

Field	Question	Answer
papskits (required)	Pap Smear Kits	1 Available<br>2 Out of Stock(< 1 month)<br>3 Out of Stock(> 1 month)<br>4 Not Available
Hpvskits (required)	HPV Screening Kits	1 Available<br>2 Out of Stock(< 1 month)<br>3 Out of Stock(> 1 month)<br>4 Not Available
acid (required)	3-5% Acetic Acid/Vinegar	1 Available<br>2 Out of Stock(< 1 month)<br>3 Out of Stock(> 1 month)<br>4 Not Available
iod (required)	Lugols Iodine	1 Available<br>2 Out of Stock(< 1 month)<br>3 Out of Stock(> 1 month)<br>4 Not Available
cwool (required)	Applicator Sticks/Cotton Wool	1 Available<br>2 Out of Stock(< 1 month)<br>3 Out of Stock(> 1 month)<br>4 Not Available
gloves (required)	Clean Gloves	1 Available<br>2 Out of Stock(< 1 month)<br>3 Out of Stock(> 1 month)<br>4 Not Available
dbucket (required)	Decontamination Bucket	1 Available<br>2 Out of Stock(< 1 month)<br>3 Out of Stock(> 1 month)<br>4 Not Available
sharps (required)	Sharps Container	1 Available<br>2 Out of Stock(< 1 month)<br>3 Out of Stock(> 1 month)<br>4 Not Available
bins (required)	Disposal Bins(Color coded)	1 Available<br>2 Out of Stock(< 1 month)<br>3 Out of Stock(> 1 month)<br>4 Not Available
Treatment of precancerous lesions		
tx (required)	Does this facility provide treatment of precancerous lesions?	1 Yes<br>0 No
Treatment		
txtype (required)	Indicate the type of treatment	1 Cryotherapy<br>2 LEEP<br>3 Thermal Ablation<br>4 Other (specify)
Treatment		
txcryo (required)	For clients due for pre-cancer treatment in your facility ,when are they treated?	1 Single Visit Approach(Same Day Treatment)<br>2 Scheduled for a later date<br>3 Referred to another facility
Treatment		
latebooking	Reasons for postponing or referral	
refer1	1	
refer2	2	
refer3	3	
refer4	4	
Status of the Cryotherapy Unit		
cunit (required)	Is the cryotherapy unit functional and in good working condition?<br>Hint:Check if cylinder with gas is available and if it has been used in the last 3 months	1 Yes<br>0 No
Status of the Cryotherapy Unit		

Field	Question	Answer
cunitbad	Why is the cryotherapy unit not functional?	

cunitstatus1	1	
cunitstatus2	2	
cunitstatus3	3	
cunitstatus4	4	
LEEP		
txleep (required)	What are the pre cancer treatment options for LEEP clients?	1 Single Visit Approach(Same Day Treatment)<br>2 Scheduled for a later date<br>3 Referred to another facility
LEEP		
leepref (required)	Where are biopsy/LEEP patients referred to?	1 Private LAB<br>2 Private referral LAB<br>3 Public referral county facility LAB<br>4 Public referral national LAB<br>5 Other
LEEP		
leepfollowup (required)	Does the facility have a follow up mechanism for referred biopsy/LEEP?	1 Yes<br>0 No
LEEP		
leepTAT	What is the turn around time for performing biopsy/LEEP in patients unsuitable for cryotherapy/thermocoagulation?	1 Less than 2 weeks<br>2 More than 2 weeks
LEEP		
leeplatebkng	Reasons for postponing or referral	
lrefer1	1	
lrefer2	2	
lrefer3	3	
lrefer4	4	
Cervical Cancer Screening & Treatment Data		
data1 (required)	Does the facility have the latest updated cervical cancer screening and treatment register?	1 Yes<br>0 No
Data		
datacap (required)	Is there an alternative way of capturing the data?<br>Hint:e.g black book	1 Yes<br>0 No
Cervical Cancer Screening & Treatment Data		
notedata	Refer to the DAR or any alternative book used to capture the data and note the summaries for the last 3 months for the following services.	
data3 (required)	Number of clients who received VIA or VIA/VILI Screening in the last 3 months	
data4 (required)	Number of clients with positive VIA or VIA/VILI result in the last 3 months	
data5 (required)	Number of clients who received HPV test in the last 3 months	
data6 (required)	Number of clients with positive HPV result in the last 3 months	
data7 (required)	Number of clients who received Pap smear in the last 3 months	
data8 (required)	Number of clients with positive cytology result in the last 3 months	
data9 (required)	Number of HIV positive clients screened in the last 3 months	
data12 (required)	Number of clients treated using cryotherapy in the last 3 months	
data14 (required)	Number of clients treated using LEEP in the last 3 months	
Infection Prevention and Control		
labelsinf	Infection &Prevention Item	1 Yes<br>0 No
water (required)	Is running water for handwash available?	1 Yes<br>0 No
dispbox (required)	Are waste disposal bag(s) available?	1 Yes<br>0 No
sops (required)	Are there standard operating procedures(SOPs)?	1 Yes<br>0 No
wipes (required)	Are hand wipes available?	1 Yes<br>0 No
Demand Generation		

Field	Question	Answer
dem (required)	Do you conduct awareness for cervical cancer services to increase demand for screen & treat services?	1 Yes<br>0 No
Demand Generation		
demand1 (required)	What activities are being conducted to increase demand for the cervical cancer screen and treat services from the community?	1 Community Outreaches<br>2 Media<br>3 Community Leaders<br>4 Churches<br>5 Community Health Workers<br>6 Cancer Awareness Months<br>7 Others
Demand Generation		
notedemand	Other activities being conducted to ensure community awareness of cervical cancer services and increase demand for those services	
reason1.1	1	
reason1.2	2	
reason1.3	3	
reason1.4	4	
Data		
jobaids (required)	Are there any cervical cancer job aids in the cervical cancer screen and treat room?	1 Yes<br>0 No
ciec (required)	Are there any cervical cancer IEC materials in and around the facility?	1 Yes<br>0 No
Demand Generation		
eduses (required)	Do you conduct educational sessions on cervical cancer?	1 Yes<br>0 No
Demand		
esf (required)	What is the frequency of the educational sessions?	1 Daily<br>2 Weekly<br>3 Monthly
Demand Generation		
healthtalks (required)	Do you conduct health talks on cervical cancer?	1 Yes<br>0 No
Demand Generation		
htf (required)	What is the frequency of the health sessions?	1 Daily<br>2 Weekly<br>3 Monthly
Demand Generation		
mobnumber (required)	On average how many women are reached during these activities each month?	
healthp (required)	Who carries out/implements health promotion activities in the facility?	1 Nurses<br>2 Public Health Officers(PHOs)<br>3 Community Health Volunteers(CHVs)<br>4 Others
Demand Generation		
notehealthp	Other health promoters used in demand generation activities	
reasonhp	1	
reasonhp1	2	
reasonhp2	3	
reasonhp3	4	
Breast Cancer Facility Assessment		
bcancer1 (required)	Does the facility offer breast cancer screening services?	1 Yes<br>0 No
Breast Cancer Facility Assessment > Breast Cancer Assessment		

Field	Question	Answer
bcancer2 (required)	What is the primary breast cancer screening test in your facility?	1 Clinical Breast Examination(CBE)<br>2 Ultrasound(US)<br>3 Mammography<br>4 Mammography and CBE(co-testing)<br>5 Mammography and US(co-testing)<br>6 CBE and US(co-testing)
bcancer3 (required)	Are the breast cancer screen-positive individuals actively contacted to ensure compliance with further assessment ?	1 Yes, systematically<br>2 No or sporadically<br>3 Unknown
bcancer3a (required)	Are the individuals with a breast cancer diagnosis actively contacted to ensure compliance with further management ?	1 Yes, systematically<br>2 No or sporadically<br>3 Unknown
mobileclinic (required)	Does the facility have a mobile clinic?	1 Yes<br>0 No
Breast Cancer Facility Assessment > Mobile Clinic		
mobilecliniqfreq (required)	How often is the mobile clinic used for community outreach for breast cancer screening?	1 Not at all<br>2 Weekly<br>3 Monthly<br>4 Sporadically
bcancerservices (required)	Does the facility provide the following services?	1 Ultrasound<br>2 Mammogram<br>3 CT-Scan<br>4 Ultra-sound guided biopsies<br>5 Histo-pathology<br>6 None
endnote		
endnote	Thank you for taking your time to complete this survey. We truly value the information you have provided. The information gathered will be used to highlight areas where we need to focus more attention in implementing cervical cancer program.	
