# Supplementary material for: Baseline assessment of cervical cancer screening and treatment capacity in 25 counties in Kenya, 2022
Source: Front Oncol. 2024 Jul 2;14:1371529. doi: 10.3389/fonc.2024.1371529 (PMC11249718; doi:10.3389/fonc.2024.1371529)
Supplement: Data Sheet 4 — Laboratory services assessment dataset codebook. [file DataSheet_4.pdf]

Cervical Cancer Lab Assessment

Field	Question	Answer
General Information		
name (required)	Name of facility incharge	
tel (required)	Telephone<br>Format: 7xxxxxxxx	
email (required)	Email	
date	Date of supervision	
superteam	Supervision Team Members(Format:Name-Designation)<br>Hint:Grace Wanjiku-Medical Officer	
super1	1	
super2	2	
super3	3	
respo.	Respondents(Format:Name-Designation)<br>Hint:Tom Oduor-Nurse	
respo1	1	
respo2	2	
respo3	3	
County (required)	Please select your County	County County
Sub (required)	Please select the name of your Sub-County	Sub Sub
Facility (required)	Please select the name of your facility	Facility Facility
Facility Information		
ftier (required)	Facility Level Tier	1 Level 1<br>2 Level 2<br>3 Level 3<br>4 Level 4<br>5 Level 5<br>6 Level 6
ownership (required)	Facility Ownership	1 GOK<br>2 Private<br>3 NGO<br>4 FBO
wrapop (required)	Facility Catchment Population(WRA)	
General		
gen1 (required)	Is this facility part of any TWG on laboratory testing?	1 Yes<br>0 No
General		
mtngfq (required)	What is the TWG meeting frequency?	1 Weekly<br>2 Monthly<br>3 Quarterly<br>4 Semi-Annually<br>5 Annually
General		
tbpartners	List the current implementing partners for HIV and TB ?	
p1	1	
p2	2	
p3	3	
p4	4	
p5	5	
p6	6	
p7	7	
stakeholdrs	List other key stakeholders in HIV and TB .<br>e.g Community Advocacy Groups	
s1	1	
s2	2	
s3	3	
s4	4	
gen4 (required)	How many staff are employed at the laboratory?	
Laboratory Processes		
labnote	This section aims to asses and monitor lab capacity and processes to identify strengths and weaknesses of the laboratory health system.	

Field	Question	Answer
genexpert (required)	Does your lab have a GeneXpert machine?	1 Yes<br>0 No
Platform Utilization		
sub1a (required)	What is the current GeneXpert utilization?<br>Example: If utilization is 50%,indicate 0.5,If 150%,indicate 1.5	
Platform Utilization		
sub1b (required)	Are all your GeneXpert modules in good working conditions?<br>Confirm when the machine was last calibrated.	1 Yes<br>0 No
sub1c (required)	Have you experienced any testing backlogs for TB?	1 Yes<br>0 No
Platform Utilization		
backlog (required)	What are the causes of the backlogs	1 Stock Outs<br>2 Machine Breakdown<br>3 High volume of samples<br>4 Other
Platform Utilization		
backlogother	List other causes of TB testing backlogs	
other1	1	
other2	2	
other3	3	
other4	4	
Platform Utilization		
testpriority (required)	How does this facility prioritize testing for TB/HIV patients?	1 Sample tested as soon as received<br>2 TB samples tested first<br>3 EID samples tested first<br>4 Other
Platform Utilization		
prio1	List other ways of prioritizing HIV/TB testing	
prio2	1	
prio3	2	
prio4	3	
prio5	4	
prionote	If the samples received are in excess of machine capacity, what do you do with the excess samples?	
excess1	1	
excess2	2	
excess3	3	
excess4	4	
Platform Utilization		
sub2c (required)	Have you experienced testing backlogs for TB/HIV?	1 Yes<br>0 No
Platform Utilization		
sub2c1 (required)	How long was the backlog?	1 Less than 1 Week<br>2 More than 1 Week
sub2d (required)	How often do high volumes of TB samples cause delays in returning Early Infant Diagnosis and Viral Load (EID/VL) test results?	1 Often(every 2 weeks)<br>2 Rare(every 2 months)<br>3 Never
sub2d1 (required)	How often do high volumes of EID/VL samples cause delays in returning TB test results?	1 Often(every 2 weeks)<br>2 Rare(every 2 months)<br>3 Never
sub2d2 (required)	How often do high volumes of POC EID samples cause delays in returning TB test results?	1 Often(every 2 weeks)<br>2 Rare(every 2 months)<br>3 Never
sub2d3 (required)	How often do machine breakdown cause delays in returning test results?	1 Often(monthly)<br>2 Rare(Quartely)<br>3 Never
sub2d4 (required)	How often do reagents stock out cause delays in returning test results?	1 Often(monthly)<br>2 Rare(Quartely)<br>3 Never
Sample Referral		

Field	Question	Answer
sample1 (required)	Does this facility refer TB samples ?	1 Yes<br>0 No
sample2 (required)	Does this facility refer EID samples ?	1 Yes<br>0 No
sample3 (required)	Does this facility refer VL samples ?	1 Yes<br>0 No
Sample Referral		
sample123a (required)	Which facility do you refer to?	
sampletmode (required)	What is the transportation mode for TB,EID and VL samples?	1 Courier Service<br>2 Riders<br>3 HCW Provider<br>4 Ambulance
transportcost (required)	Who pays for sample transportation?	1 Facility<br>2 County<br>3 Implementing Partner
sample6	What challenges do you face in sample transportation?	
sample7	1	
sample8	2	
sample9	3	
sample10	4	
sample11	5	
Data		
data1.1 (required)	Does this facility have an electronic medical records system?	1 Yes<br>0 No
Data		
esystemlist (required)	Specify the system	1 Kenya EMR<br>2 IQ Care<br>3 Other
Data		
esystemother	List any other electronic medical records system in use	
emr1	1	
emr2	2	
emr3	3	
emr4	4	
emr5	5	
Data		
inter (required)	Does the facility have internet?	1 Yes<br>0 No
Data		
wifi (required)	How is internet provided within the lab ?	1 Fiber<br>2 Phone Tethering<br>3 Modem<br>4 WI-FI Hotspot
Commodity		
comm1 (required)	Is there a register to track HPV /Pap Smear screening commodities?	1 Yes<br>0 No
comm3 (required)	Have you ever experienced any stock out of the above mentioned commodities ?	1 Yes<br>0 No
Commodity		
stockoutperiod (required)	What was the duration of the stock-out?	1 Less than 1 Month<br>2 1 Month<br>3 More than 1 Month
stockrecord (required)	How did you report for the stock outs?	1 LIMS System<br>3 Call partners<br>4 Call County Medical Lab.Techs
stock7 (required)	How did you resolve the stockouts?	1 Waited for the next scheduled shipment<br>2 Buffer Stock from Partners<br>3 Other
Commodity		

Field	Question	Answer
notecommodity	List other ways you were able to resolve the stockouts	
commo1	1	
commo2	2	
commo3	3	
commo4	4	
commo5	5	
